# Machine Learning in Dementia Research: Trends, Biomarkers, Prediction Goals, and Data Modalities

**DOI:** 10.1002/brb3.71847

**Published:** 2026-10-15

**Authors:** Yao‐Chin Wang, Hsien Chang Yeh, Md. Mohaimenul Islam, Woon‐Man Kung

**Affiliations:** ^1^ Department of Emergency Medicine Taipei Medical University Hospital Taipei Taiwan; ^2^ Graduate Institute of Injury Prevention and Control, College of Public Health Taipei Medical University Taipei Taiwan; ^3^ School of Medicine, College of Medicine Taipei Medical University Taipei Taiwan; ^4^ Division of Outcomes and Practice Advancement, Department of Pharmacy Practice, School of Pharmacy and Pharmaceutical Sciences University At Buffalo Buffalo New York USA; ^5^ Division of Neurosurgery, Department of Surgery, Taipei Tzu Chi Hospital Buddhist Tzu Chi Medical Foundation New Taipei City Taiwan; ^6^ Interdisciplinary Program Kinesiology and Health Chinese Culture University Taipei Taiwan

**Keywords:** Alzheimer's disease, bibliometric analysis, deep learning, dementia, machine learning

## Abstract

**Objective:**

Machine learning (ML) is increasingly used in dementia research for diagnosis, early detection, prognosis, biomarker characterization, and disease phenotyping. This study aimed to provide a bibliometric and thematic overview of ML applications in dementia, emphasizing biological mechanisms, prediction goals, data modalities, collaborative networks, and emerging computational approaches.

**Methods:**

A title‐field search of the Web of Science Core Collection identified 3,833 records related to ML and dementia. After restricting publications to 2000 onward and excluding non‐analytic document types, 3,479 records were included in the bibliometric analysis. A subset of 3,223 original articles was used for thematic analyses. Publication trends, authorship, institutional collaboration, keywords, biomarker themes, ML objectives, data modalities, methods, shared research resources, and large language model (LLM) applications were evaluated.

**Results:**

ML‐related dementia research increased substantially over time. Diagnostic classification was the predominant ML objective (55.0%), followed by early detection or screening (10.3%), progression or cognitive‐decline prediction (6.8%), and biomarker or feature discovery (4.7%). Structural magnetic resonance imaging was the most frequently identified modality (42.6%), followed by clinical or cognitive data (22.4%), positron emission tomography/single photon emission computerized tomography (10.3%), and genetic or genomic data (10.2%). Prominent biological themes included hippocampal measures, amyloid/Aβ, electroencephalogram, tau/p‐tau, and cerebrospinal fluid biomarkers. Institutional networks showed distinct international collaboration clusters and frequent use of shared infrastructures. Transformer‐based and LLM approaches emerged in recent years.

**Conclusions:**

ML research in dementia has expanded beyond diagnostic neuroimaging toward multimodal, biomarker, prognostic, and language model applications, although diagnostic classification and structural imaging remain dominant. Emphasis on biological integration, longitudinal validation, and clinical translation are needed.

## Graphical abstract

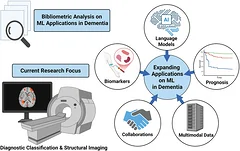

## 1 Introduction

Dementia is a neurodegenerative condition characterized by progressive deterioration in memory, cognition, behavior, and the ability to perform daily activities, affecting about 55 million people globally (Błaszczyk 2022; Fymat, 2019; Malik et al., 2022). The number of people living with dementia is projected to increase substantially by 2050 as populations age, with particularly rapid growth expected in low and middle‐income countries (Paul and Nag; Kowal et al., 2024). Alzheimer's disease is the most common form of dementia, accounting for approximately two‐thirds of cases (Association, 2017). Dementia also imposes considerable emotional and financial burdens on patients, families, caregivers, and healthcare systems (Papastavrou et al., 2007; Stokes et al., 2014). The global economic burden already exceeds one trillion United States (US) dollars annually and is expected to increase as dementia prevalence rises (Nandi et al., 2024; Brück et al., 2023). These trends highlight the need for earlier detection, improved understanding of disease mechanisms, more effective risk stratification, and development of preventive and therapeutic strategies.

Dementia research, including diagnosis and patient care, has increasingly incorporated machine learning (ML) and other artificial intelligence (AI) approaches to analyze complex and high‐dimensional biomedical data (Moya, 2024). ML methods can integrate information from structural and functional neuroimaging, genetic and genomic data, cognitive assessments, electronic clinical information, and biological biomarkers, potentially enabling detection of disease‐related patterns before overt clinical manifestations become apparent (Vrahatis et al., 2023, Bettencourt et al., 2023; Cruz et al., 2023). In Alzheimer's disease and related dementias, growing attention has been directed toward biologically informative markers, such as amyloid and tau pathology, neuroimaging‐derived features, genetic susceptibility, and fluid biomarkers (Khan and Iturria‐Medina, 2024; Lin et al., 2025). ML provides a computational framework for examining relationships among these heterogeneous signals and clinical phenotypes (DeGroat et al., 2024). In addition to diagnostic classification, ML models have been developed for early detection, prediction of progression from mild cognitive impairment to dementia, cognitive decline forecasting, disease subtyping, biomarker identification, image segmentation, and individualized risk prediction (Shaikh et al., 2025). ML may also support drug discovery by identifying potential therapeutic targets or candidate compounds (Niazi and Mariam, 2023), while AI‐enabled monitoring and assistive technologies may contribute to patient support and caregiving (Aminizadeh et al., 2024). Consequently, understanding not only which ML algorithms are being used, but also the clinical questions they address and the data modalities on which they depend, is increasingly important for evaluating the direction of the field.

Bibliometric analysis provides a quantitative framework to access collaboration patterns, emerging themes, influential contributors, and research growth (Donthu et al., 2021). Co‐authorship, citation, keyword analyses, and publication may identify broader research directions (Wu et al., 2023). Data modalities related to ML use in dementia, limited insights in biological topics, and prediction goals were provided by previous bibliometric studies. While modern large language model (LLM) and transformer‐based approaches can seize contextual relationships, conventional keyword co‐occurrence methods may fail to recognize them. We aimed to provide an updated bibliometric and thematic assessment of ML applications in dementia, such as biomarkers, collaborations, data modalities, emerging language model applications, prediction goals, and publication trends.

## 2 Material and Methods

### 2.1 Data Source

A title‐field search was conducted on the Web of Science (WoS) Core Collection to identify publications related to ML applications in dementia. The search covered publications through September 1, 2026. WoS was selected because it provides standardized bibliographic information suitable for evaluating publication trends, citation patterns, authorship, institutional affiliations, and research networks (Pranckutė, 2021; López‐Illescas et al., 2008). It is an excellent resource for bibliometric evaluation and is chosen for its quality records. It is supported by detailed examination of publication patterns, citation metrics, and research impact within ML and dementia. A solid foundation for evaluating research activity from WoS data allows accurate assessment of trends and development of ML applications for dementia.

### 2.2 Inclusion and Exclusion Criteria

Eligible publications included English‐language studies that address ML or AI applications in dementia. Our study excluded non‐analytic document articles, corrections, editorials, letters, meeting abstracts, and news items. Publications before 2000 were also excluded. 3,479 records were included in the overall bibliometric analysis after these exclusions. We further restricted the dataset to 3,223 original research publications to avoid treating review articles as primary ML applications for analysis of ML prediction goals, data modalities, biomarkers, disease mechanisms, and computational methods.

### 2.3 Data Extraction and Thematic Classification

Bibliographic metadata were extracted from WoS and combined across all exported files using unique WoS identifiers. Titles, abstracts, author keywords, and Keywords Plus were used for thematic classification. A predefined, rule‐based taxonomy was developed to identify biological mechanisms and biomarkers, ML prediction goals, data modalities, and computational methods.

Biological themes included amyloid and tau pathology, neuroinflammation, vascular mechanisms, synaptic dysfunction, and other disease‐related processes. Biomarker categories included amyloid/Aβ, tau/p‐tau, apolipoprotein E (APOE), cerebrospinal fluid and blood biomarkers, hippocampal measures, electroencephalogram (EEG), and other biological or digital signals. Data modalities included structural and functional magnetic resonance imaging (MRI), positron emission tomography (PET)/single photon emission computerized tomography (SPECT), EEG/magnetoencephalography (MEG), clinical and cognitive assessments, genetic/genomic data, fluid biomarkers, speech or language data, and digital or wearable measures. Multiple biomarker and data‐modality categories could be assigned to the same publication.

ML objectives were classified according to the principal research question, including diagnostic classification, early detection or screening, disease progression prediction, cognitive decline prediction, conversion prediction, disease subtyping or phenotyping, biomarker or feature discovery, image segmentation, and other predictive applications.

We developed taxonomy before conducting the final thematic analyses to ensure that publications were classified consistently. The domains and categories were guided by previous evidence on ML in dementia, including studies of biomarkers, neuroimaging, genetics and genomics, and disease prediction. Finally, we polished the taxonomy by assessing commonly used terms throughout the titles, abstracts, author keywords, and Keywords Plus in the retrieved publications. Before classification, we defined each category and specified relevant terms, abbreviations, and synonyms. Publications were assigned to more than one category for biological mechanisms, biomarkers, data modalities, and computational methods, whereas each publication was assigned one primary ML research objective. The complete taxonomy, including category definitions and classification rules, is provided in Table S1.

### 2.4 Taxonomy Development and Validation

To examine the content validity and coverage of the taxonomy, two reviewers who are experts in dementia research and ML reviewed the category definitions, terminology, and areas of potential overlap. They focus on whether the taxonomy sufficiently captured the biological mechanisms, biomarkers, data modalities, computational approaches, and research objectives presented in collected literature. Ambiguous or overlapping definitions were examined and resolved by consensus before the classification rules were established.

We evaluated classification performance in a stratified sample of 120 original research articles, selecting 10 articles from each primary research goal category. The same two reviewers independently classified each article using the title, abstract, and keywords and were blinded to the rule‐based assignments. We resolved disagreements by consensus and used the consensus classification as the reference standard. We assessed agreement between the rule‐based and manual classifications for the primary research goal using percent agreement and Cohen κ.

### 2.5 Bibliometric Analysis

Temporal trends, co‐authorship networks, keyword co‐occurrence patterns, country‐level clusters, and institutional collaborations were analyzed. Visualization of Similarities Viewer was used to visualize conventional bibliometric networks. Keyword analysis incorporated author keywords and keywords Plus, with common spelling variants and related terminology standardized before analysis. Keywords occurring at least 15 times were retained for network visualization.

Annual publication output was calculated to characterize the growth of ML‐related dementia research. Because the search was conducted on September 1, 2026, publication counts for 2026 were considered incomplete and were interpreted separately from complete calendar years.

The annual growth rate of publications was calculated using the following formula:

$$\mathrm{Annual}\;\mathrm{Growth}\;\mathrm{Rate}\;\;\left(\%\right)=\left(\frac{\mathrm{Number}\;\mathrm{of}\;\mathrm{Publications}\;\mathrm{in}\;\mathrm{Current}\;\mathrm{Year}-\mathrm{Number}\;\mathrm{of}\;\mathrm{Publication}\;\mathrm{ofPrevious}\;\mathrm{Year}}{\mathrm{Number}\;\mathrm{of}\;\mathrm{Publication}\;\mathrm{of}\;\mathrm{Previous}\;\mathrm{Year}}\right)\;\times 100$$

This formula assesses the annual changes in publication activity and provides a clear picture of shifts in research ideas related to ML applications in dementia.

Co‐authorship relationships were evaluated according to the number of publications shared by two authors. Node size represented publication output, whereas connecting lines represented co‐authorship, and greater link thickness indicated stronger collaboration.

Institutional collaboration was analyzed using standardized WoS affiliation information. Institutions with at least five publications were included in the collaboration network, and connections were weighted according to the number of shared publications. Major institutional communities were further characterized according to their leading institutions and cross‐cluster collaborations. References to commonly used multicenter datasets or research infrastructures, including Alzheimer's Disease Neuroimaging Initiative (ADNI) and other dementia‐related resources, were identified from publication metadata. Such mentions were interpreted as evidence of association with shared research infrastructures rather than proof of direct patient‐level data sharing between institutions.

To extend conventional bibliometric analysis beyond keyword frequency, additional thematic analyses examined relationships among biological markers, ML objectives, data modalities, and computational methods. Cross‐tabulations were generated for ML goal by data modality, ML method by prediction goal, and ML method by data modality. Temporal trends in major biomarker themes were also examined.

Recent applications of transformer‐based models and LLMs were identified separately to characterize their emergence within dementia research. Conventional keyword co‐occurrence analysis remained the primary bibliometric approach because of its transparency and reproducibility; transformer‐ and LLM‐based semantic approaches were considered complementary methods capable of capturing contextual relationships that may not be reflected by exact keyword overlap.

## 3 Results

### 3.1 Publication Outputs and Study Characteristics

The WoS search identified 3,833 unique records related to ML applications in dementia research. After excluding 20 publications published before 2000 and 334 non‐analytic document types, 3,479 records were retained for the bibliometric analysis. A subset of 3,223 original research publications was used for thematic analyses of biological mechanisms, biomarkers, ML prediction goals, data modalities, and computational methods (Figure 1A).

**FIGURE 1:**
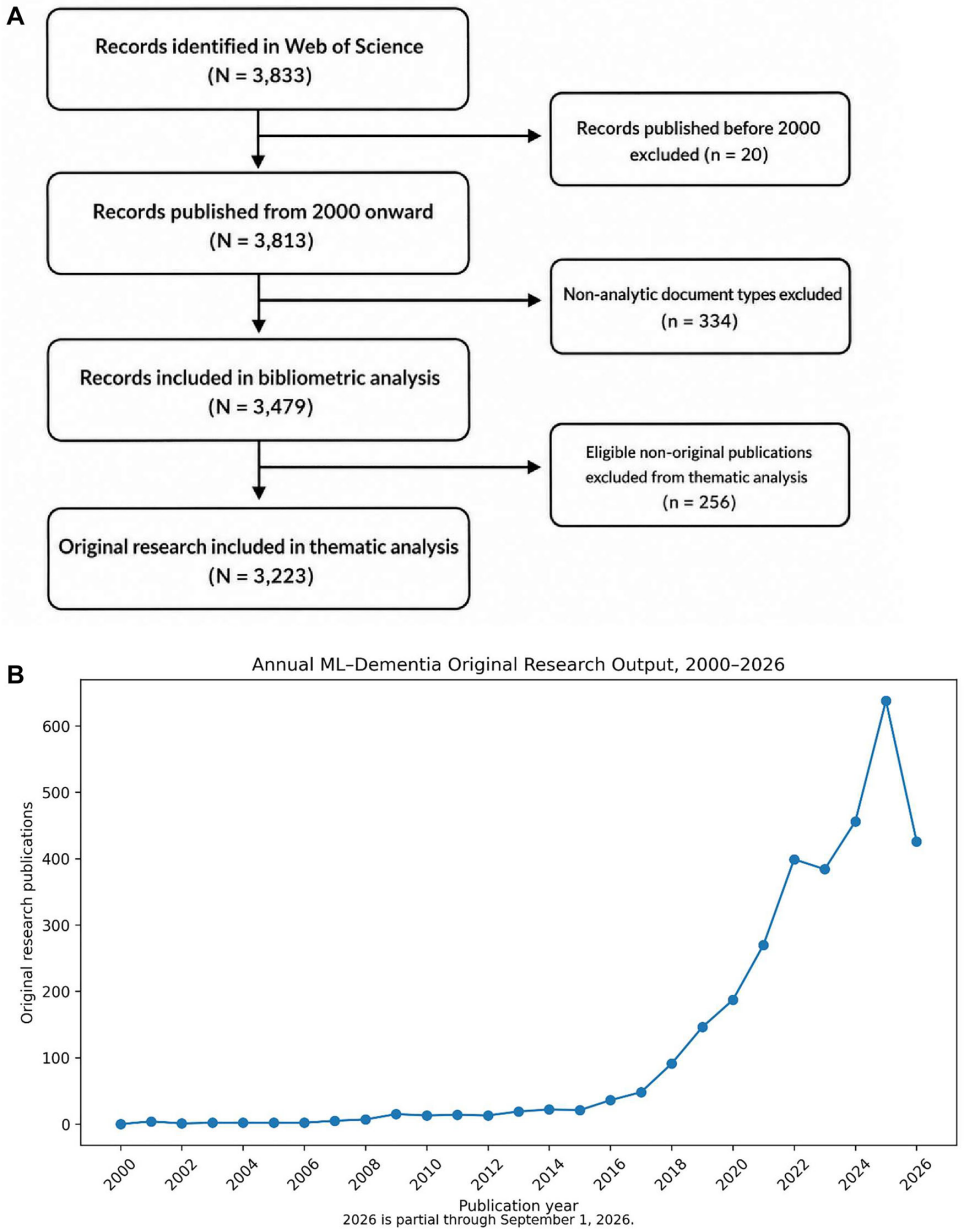
Study selection and publication trends. A. Screening flow chart. B. Annual publication output.

Research output increased substantially over time (Figure 1B). Annual eligible publications increased from fewer than 100 per year before 2018 to 204 in 2020, 420 in 2022, 493 in 2024, and 694 in 2025, the highest complete publication year. An additional 471 eligible publications were identified in 2026 through September 1; therefore, 2026 was considered a partial year. Conventional bibliometric characteristics, including the leading journals, countries, authors, institutions, and highly cited publications, are presented in Tables S2–S5.

### 3.2 Biological Mechanisms and Biomarker Themes

Among the 3,223 original research publications, amyloid pathology was the most frequently identified biological mechanism (*n =* 290, 9.0%), followed by tau pathology (*n =* 184, 5.7%), neuroinflammation/immune mechanisms (*n =* 95, 2.9%), vascular/cerebrovascular mechanisms (*n =* 78, 2.4%), oxidative/mitochondrial mechanisms (*n =* 52, 1.6%), and synaptic dysfunction (*n =* 45, 1.4%) (Figure 2A). Most mechanistic publications were concentrated from 2021 to 2026.

**FIGURE 2:**
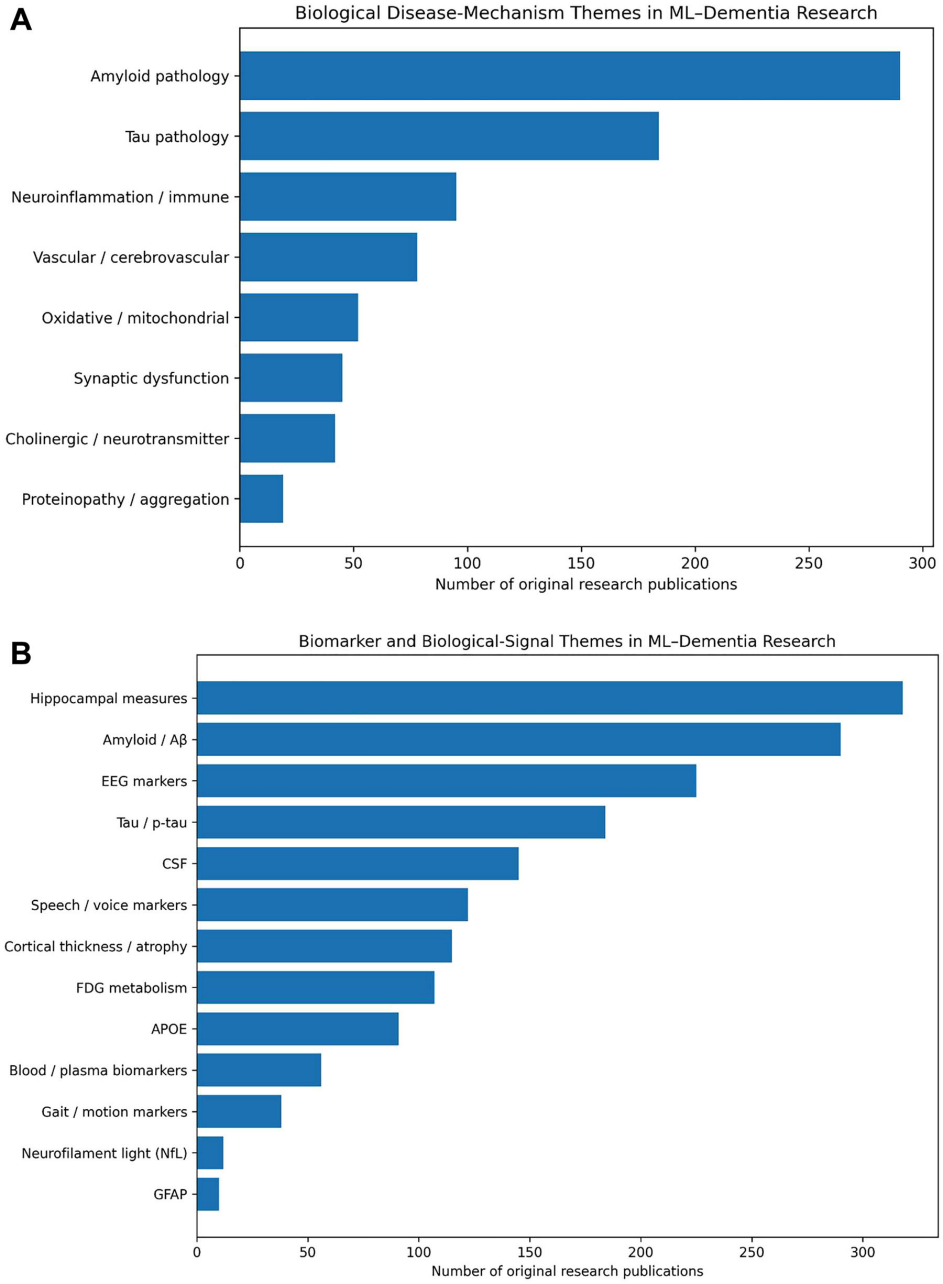
Biological and biomarker landscape. A. Biological disease‐mechanism themes. B. Biomarker and biological signal themes.

Biomarker analysis showed substantial use of hippocampal measures (*n =* 318, 9.9%), amyloid/Aβ (*n =* 290, 9.0%), EEG‐derived markers (*n =* 225, 7.0%), tau/p‐tau (*n =* 184, 5.7%), and cerebrospinal fluid (CSF) biomarkers (*n =* 145, 4.5%) (Figure 2B). Other themes included speech/voice markers (3.8%), cortical thickness or atrophy (3.6%), fluorodeoxyglucose (FDG) metabolism (3.3%), and APOE (2.8%). Blood/plasma biomarkers, neurofilament light, and Glial Fibrillary Acidic Protein (GFAP) appeared predominantly in the most recent publication period. Annual trends for individual biomarkers are provided in Figure S1.

### 3.3 ML Prediction and Research Goals

Diagnostic classification remained the dominant ML objective, accounting for 1,774 studies (55.0%) (Table 1; Figure 3A). This was followed by early detection or screening (*n =* 332, 10.3%), progression or cognitive decline prognosis (*n =* 218, 6.8%), biomarker or feature discovery (*n =* 151, 4.7%), and segmentation or image quantification (*n =* 113, 3.5%).

**TABLE 1:** Primary machine learning prediction and research goals in dementia studies.

Primary ML prediction/research goal	N (%) of original research*	2000–2015	2016–2020	2021–2026
Diagnostic classification	1,774 (55.0)	94	310	1,370
Early detection/screening	332 (10.3)	8	39	285
Progression/cognitive decline prognosis	218 (6.8)	3	27	188
Biomarker/feature discovery	151 (4.7)	5	18	128
Segmentation/image quantification	113 (3.5)	4	12	97
Risk/incident dementia prediction	94 (2.9)	0	11	83
Treatment/drug discovery/response prediction	89 (2.8)	1	8	80
Subtyping/heterogeneity/phenotyping	88 (2.7)	1	11	76
MCI conversion prediction	83 (2.6)	5	17	61
Severity/staging	55 (1.7)	4	8	43
Monitoring/digital phenotyping	47 (1.5)	4	12	31
Other/unclear	179 (5.6)	13	35	131
**Total**	3,223 (100.0)	142	508	2,573

Abbreviations: MCI, mild cognitive impairment, ML, machine learning, N, number.

**FIGURE 3:**
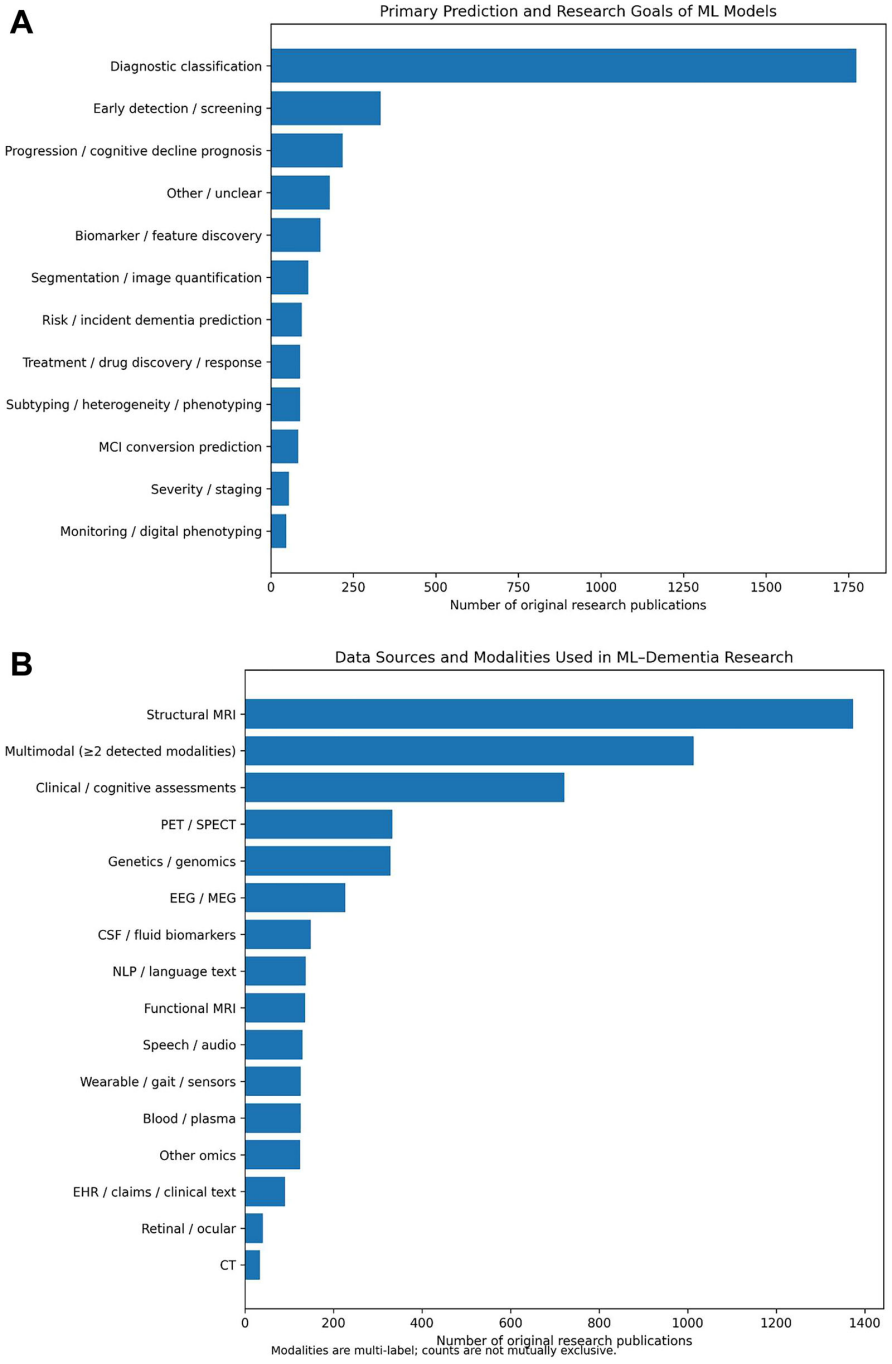
Scientific objectives and data sources. A. Primary machine learning prediction/research goals. B. Data modalities used in machine learning studies.

Other research objectives included incident dementia risk prediction (*n =* 94, 2.9%), treatment or drug‐response applications (*n =* 89, 2.8%), disease subtyping or phenotyping (*n =* 88, 2.7%), and mild cognitive impairment (MCI) conversion prediction (*n =* 83, 2.6%). Smaller proportions addressed disease staging and digital monitoring.

These findings indicate that although diagnostic classification remains predominant, ML is increasingly being used to address prognosis, disease heterogeneity, biomarker discovery, and longitudinal prediction.

### 3.4 Data Modalities and Computational Methods

Structural MRI was the most frequently identified data modality (*n =* 1,374, 42.6%), followed by clinical or cognitive assessments (*n =* 722, 22.4%), PET/SPECT (*n =* 333, 10.3%), and genetics/genomics (*n =* 329, 10.2%) (Figure 3B). EEG/MEG was identified in 7.0% of studies, while CSF/fluid biomarkers, functional MRI, speech/audio, natural language processing/text data, blood/plasma biomarkers, wearable/sensor data, and other omics each accounted for smaller but increasingly visible proportions. Multimodal approaches were identified in 1,013 studies (31.4%).

The most common computational approach was the convolutional neural network (CNN) (*n =* 838, 26.0%), followed by support vector machines (*n =* 530, 16.4%), random forests (*n =* 417, 12.9%), transformer/LLM‐related methods (*n =* 327, 10.1%), transfer learning (*n =* 267, 8.3%), and gradient boosting/Extreme Gradient Boosting (*n =* 266, 8.3%).

Cross‐analysis of ML goals and data modalities demonstrated that diagnostic classification was strongly associated with structural MRI and other neuroimaging data, whereas progression, biomarker discovery, and disease‐subtyping studies showed greater use of clinical, genetic, biomarker, and multimodal data (Figure 4). Detailed method‐by‐goal and method‐by‐modality matrices are provided in Figures S2 and S3.

**FIGURE 4:**
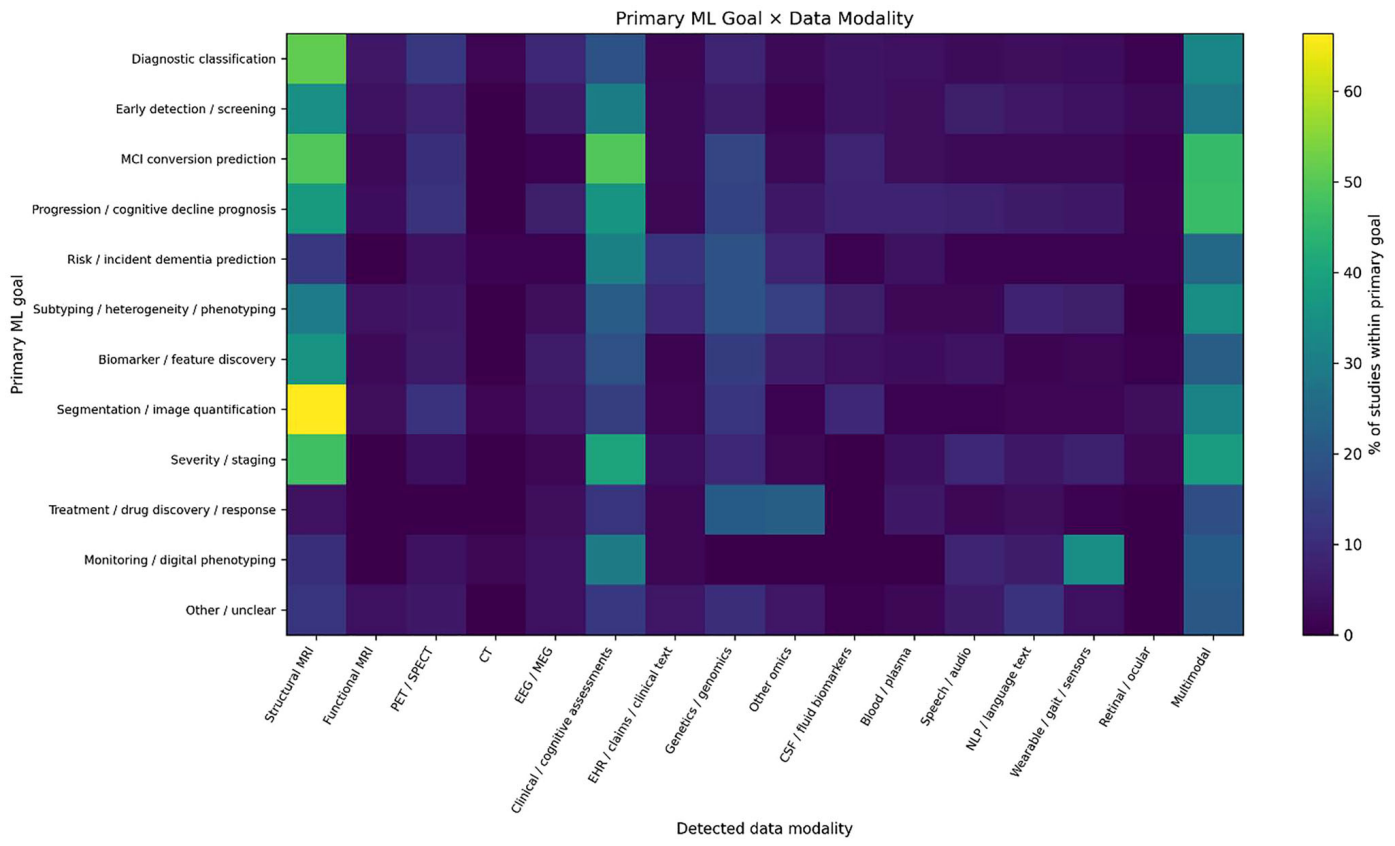
Machine learning prediction goal × data modality heatmap.

### 3.5 Institutional Collaboration and Shared Research Infrastructure

Institutional network analysis identified nine collaboration clusters comprising 587 institutions (Table 2; Figure 5). The largest cluster included 156 institutions and reflected a China/Asia‐led international computational and imaging network. Other major clusters represented the United Kingdom (UK)–European neurodegeneration collaborations, South Asia–Middle East–Korea computational collaborations, and US academic medical center collaborations.

**TABLE 2:** Major institutional collaboration clusters and shared research resources in machine learning‐based dementia research.

Panel A. Major institutional collaboration clusters.					
Cluster	Institutions, N	Cluster‐associated publications, N	Collaboration profile	Leading institutions	Most frequently identified datasets/consortia
**Cluster 1**	156	907	China/Asia‐led international computational and imaging collaboration	Chinese Academy of Sciences; Vellore Institute of Technology; Capital Medical University; Shanghai Jiao Tong University; Fudan University	ADNI (215); OASIS (26); UK Biobank (16); NACC (10); AIBL (9)
**Cluster 2**	130	591	UK–European neurodegeneration collaboration	University of London; University College London; King's College London; University of Cambridge; Inserm	ADNI (120); OASIS (20); UK Biobank (10); AIBL (9); NACC (6)
**Cluster 3**	128	738	South Asia–Middle East–Korea computational collaboration	Egyptian Knowledge Bank; National Institute of Technology System; Korea University; SRM Institute of Science and Technology; King Abdulaziz University	ADNI (197); OASIS (65); AIBL (7); NACC (3); MIRIAD (3)
**Cluster 4**	124	681	US academic medical center collaboration	University of California System; Harvard University; Harvard Medical School/Affiliates; University of Pennsylvania; State University System of Florida	ADNI (138); OASIS (21); NACC (15); Framingham Heart Study (9); UK Biobank (9)

Panel B. Most frequently identified shared datasets and research consortia.				
Dataset/consortium	Publications with metadata mention, N (%) of bibliometric corpus*	2000–2015	2016–2020	2021–2026
ADNI	771 (22.2)	21	133	617
OASIS	197 (5.7)	3	24	170
UK Biobank	33 (0.9)	0	0	33
AIBL	32 (0.9)	0	4	28
NACC	26 (0.7)	0	3	23

*Note*: *Percentages for shared datasets/consortia are based on the 3,479‐publication bibliometric corpus. Institutional clusters were derived from co‐authorship relationships among institutions meeting the network inclusion threshold. Cluster labels provide an interpretation of the dominant geographic and institutional composition rather than formal consortium names. Dataset or consortium mentions indicate association with shared research infrastructure and should not be interpreted as evidence of direct patient‐level data sharing between institutions.

Abbreviations: ADNI, Alzheimer's disease neuroimaging initiative; AIBL, Australian imaging, biomarkers and lifestyle study; MIRIAD, minimal interval resonance imaging in Alzheimer's disease; N, number; NACC, national Alzheimer's coordinating center; OASIS, open access series of imaging studies; UK, United Kingdom; US, United States.

**FIGURE 5:**
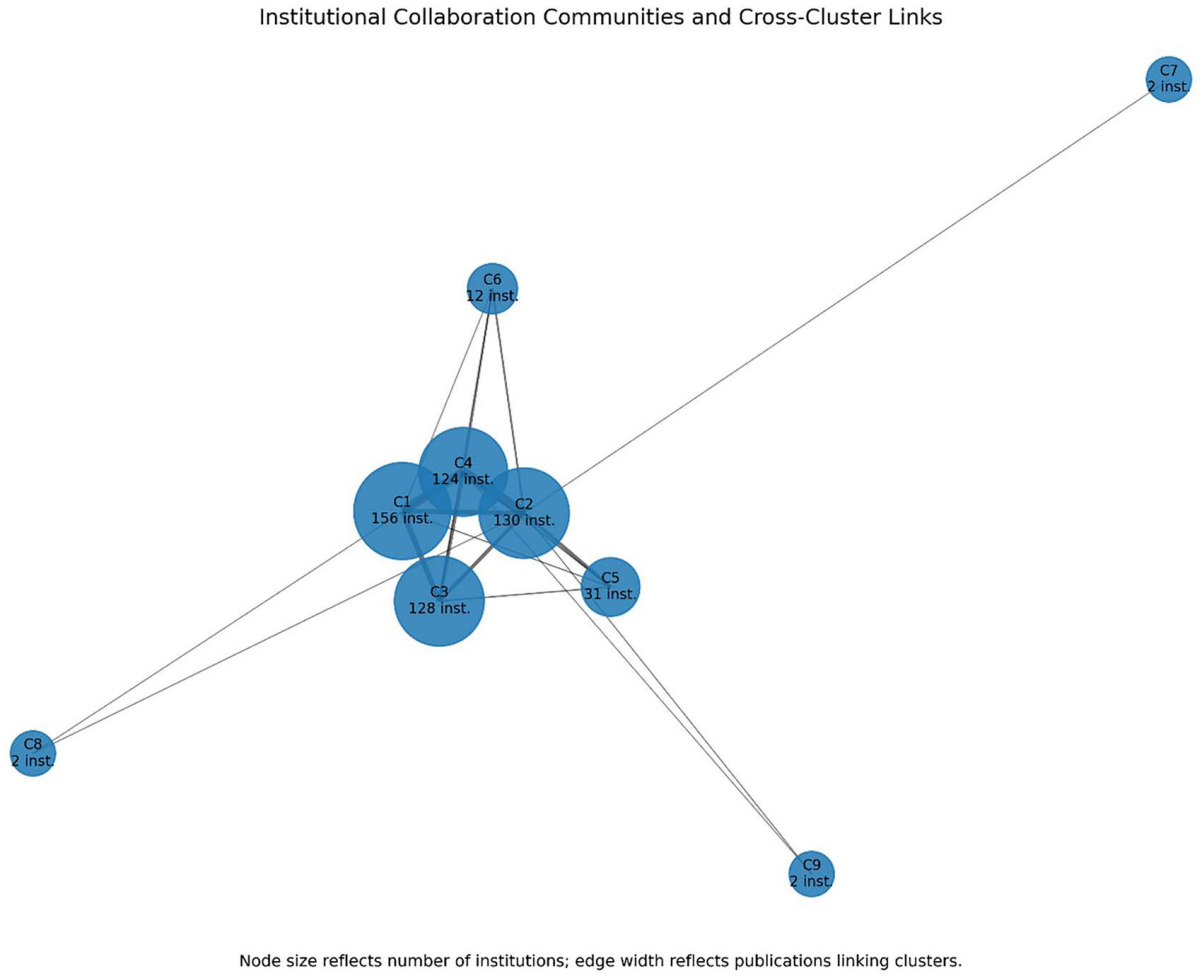
Institutional collaboration network.

The strongest cross‐cluster collaboration was observed between the UK–European and US clusters, with 135 publications linking the two communities, followed by the China/Asia and US clusters with 121 linking publications.

The institutional structure was also associated with extensive use of shared dementia research resources. ADNI was identified in 771 publications (22.2% of the bibliometric corpus), substantially more than open access series of imaging studies (OASIS) (*n =* 197, 5.7%), UK Biobank (*n =* 33), Australian Imaging, Biomarkers and Lifestyle Study (AIBL) (*n =* 32), and National Alzheimer's Coordinating Center (NACC) (*n =* 26) (Supplementary Figure S4). These associations indicate the importance of shared research infrastructure in supporting multi‐institutional ML research but do not necessarily demonstrate direct patient‐level data sharing between collaborating institutions.

### 3.6 Keyword Structure and Emerging LLM Research

Keyword analysis identified five broad thematic areas: clinical risk and biomarkers; deep learning and neuroimaging; MCI conversion and progression; diagnostic criteria and staging; and frontotemporal dementia/differential diagnosis. Biomarker‐related terminology, including amyloid, tau, CSF biomarkers, hippocampal atrophy, genetics, and FDG‐PET, appeared across several clusters rather than forming an isolated biological theme. Detailed keyword‐cluster results are provided in Table S6.

Transformer‐family and LLM approaches emerged primarily in recent years. Transformer‐related publications increased from 2 in 2020 to 92 in 2025, while explicit LLM/generative AI publications increased from 1 in 2022 to 29 in 2025 (Figure S5).

## 4 Discussion

### 4.1 Public Health Implications

Dementia remains a major public health challenge because of its progressive course, substantial effects on patients and caregivers, and growing healthcare burden (Islam et al., 2017, Cohen and Eisdorfer, 2001). Our updated analysis demonstrates a rapid expansion of ML research in dementia, particularly since 2020. ML applications now extend beyond conventional diagnostic classification to early detection, prognosis, biomarker discovery, disease subtyping, treatment‐related research, and digital monitoring. These developments highlight the increasing role of computational methods in analyzing complex dementia‐related data and potentially supporting earlier and more individualized clinical decision‐making (Kalani and Anjankar, 2024).

### 4.2 Biological Themes, Prediction Goals, and Data Modalities

A major finding of this study is the growing integration of ML with biological and biomarker research. Amyloid and tau pathology were the most prominent disease‐mechanism themes (Shaikh et al., 2025, Shukla and Singh, 2022, Winchester et al., 2023, Wang et al., 2024), while hippocampal measures, amyloid/Aβ, EEG‐derived signals, tau/p‐tau, and CSF biomarkers were frequently represented (Bettencourt et al., 2023, Rossini et al., 2022, Campagner et al., 2025). More recent studies also incorporated blood‐based biomarkers, neurofilament light, and GFAP. These findings suggest that ML research is increasingly moving beyond purely clinical classification toward characterization of biological processes underlying dementia (Shaikh et al., 2025, Winchester et al., 2023).

Nevertheless, diagnostic classification remained the dominant ML objective, accounting for more than half of original research studies. Data modalities showed a similar pattern. Structural MRI remained the most frequently used source, reflecting the strong historical relationship between neuroimaging and ML in dementia research (Borchert et al., 2023, Aghdam et al., 2025, Grigas et al., 2024). However, approximately one‐third of studies incorporated multiple detected modalities, and clinical/cognitive information, PET, genetics, fluid biomarkers, EEG, speech, and sensor‐derived measures were increasingly represented. Combining these complementary sources may provide more comprehensive representations of dementia than single modality models.

### 4.3 ML Methods and Emerging Language Models

CNNs related to neuroimaging‐based diagnostic applications were the most widely identified computational approaches. Frequently used methods also included gradient boosting methods, random forests, support vector machines, and transfer learning. Since different ML methods resolve different research questions instead of serving interchangeable aims, the relationship between algorithm choice and data modality is relevant.

In recent years, LLM and transformer‐based approaches have surfaced. The growth represents a surge of dementia ML research toward clinical text, context‐sensitive analysis, language, and multimodal information. To identify the overlook of keyword co‐occurrence, LLMs may enhance traditional bibliometric techniques. Before the applications can be routinely incorporated into clinical or bibliometric research, they require attention to reproducibility, careful validation, and transparency (Gong et al., 2026, Heyat et al., 2026).

### 4.4 Institutional Collaboration and Shared Research Infrastructure

Some huge global collaboration communities involving institutions from North America, Europe, Asia, and other regions were noticed by the affiliated network. Importantly, these networks were frequently associated with large, shared dementia research infrastructures. ADNI was by far the most identified resource, followed by OASIS, UK Biobank, AIBL, and NACC. Access to large, standardized datasets likely facilitates development and comparison of ML models across institutions and may partly explain the dense collaboration networks observed.

However, co‐authorship or use of the same research resource should not be interpreted as evidence that institutions directly exchanged patient‐level data. Future collaboration networks may benefit from broader geographic representation and greater participation from populations and healthcare systems currently underrepresented in dementia ML research.

### 4.5 Strengths and Limitations

Our study provides an updated bibliometric analysis while extending conventional publication and keyword analyses to examine biological mechanisms, biomarkers, ML prediction goals, data modalities, computational methods, institutional collaboration, shared research resources, and emerging LLM applications. This broader approach provides greater scientific context regarding how ML is actually being used in dementia research.

Several limitations should be considered. First, the study relied exclusively on WoS, and relevant publications indexed only in other databases may therefore have been missed. Second, the literature was identified using a title‐field search, which improves specificity but may exclude studies in which dementia or ML terminology appears only in the abstract or keywords. Third, thematic classifications were derived from bibliographic metadata and automated rule‐based approaches and may not capture every methodological detail reported in the full text. Finally, bibliometric associations describe publication patterns and research activity but cannot determine model quality, clinical effectiveness, external validity, or causal relationships.

### 4.6 Future Directions

Future ML research in dementia should move beyond predominantly cross‐sectional diagnostic classification toward longitudinal prediction, biological characterization, disease subtyping, multimodal integration, and clinically actionable decision support. Greater incorporation of blood and molecular biomarkers, longitudinal clinical data, diverse populations, and external validation will be important for improving generalizability and clinical relevance. Explainable and reproducible ML approaches should also remain priorities as more complex deep learning and language model methods are introduced.

## 5 Conclusions

Our cross‐sectional study shows ML research in dementia has expanded rapidly and is becoming increasingly interdisciplinary. Although diagnostic classification and structural neuroimaging remain dominant, the field is evolving toward multimodal data integration, biological biomarkers, prognostic modeling, collaborative research infrastructures, and emerging transformers and LLM applications. These developments provide important opportunities to improve biological understanding, prediction, and ultimately clinical management of dementia.

## Author Contributions

**Yao‐Chin Wang**: conceptualization, funding acquisition, methodology, project administration, writing‐ original draft. **Hsien Chang Yeh**: data curation, validation, visualization. **Md. Mohaimenul Islam**: formal analysis, investigation, resources, software. **Woon‐Man Kung**: supervision, validation, writing‐ reviewing and editing. All authors read and approved the final manuscript.

## Funding

This research was funded by the National Science and Technology Council, Taiwan, grant number 115‐2320‐B‐038‐072‐MY2.

## Conflicts of Interest

The authors declare no conflicts of interests.

## Supporting information




**Supplementary Figure**: brb371847‐sup‐0001‐SuppMat.docx

## Data Availability

Data will be made available on request.
