# Supplementary material for: Machine Learning in Dementia Research: Trends, Biomarkers, Prediction Goals, and Data Modalities
Source: Brain Behav. 2026 Oct 15;16(10):e71847. doi: 10.1002/brb3.71847 (PMC13673231; doi:10.1002/brb3.71847)
Supplement: Supplementary file 1 [file BRB3-16-e71847-s001.docx]

**Supplementary Tables and Figures**

**Supplementary Tables**

**Table S1. Operational Taxonomy and Text-Matching Rules Used for Thematic Classification**

| **Domain** | **Category** | **Operational definition** | **Text signals/terms** | **Assignment** |
| --- | --- | --- | --- | --- |
| Biological mechanism | Amyloid pathology | Studies addressing amyloid-related disease biology | amyloid, amyloid beta, Aβ, ABeta | Multi-label |
| Biological mechanism | Tau pathology | Studies addressing tau-related pathology | tau, p-tau, phosphorylated tau, tauopathy | Multi-label |
| Biological mechanism | Neuroinflammation/  immune | Inflammatory or immune mechanisms | neuroinflammation, microglia, astrocyte, cytokine, inflammation | Multi-label |
| Biomarker/signal | APOE | APOE-related biological or predictive information | APOE, apolipoprotein E | Multi-label |
| Data modality | Structural MRI | Structural MRI-derived information | MRI, structural MRI, T1-weighted, DTI, VBM | Multi-label |
| ML method | CNN | Convolutional neural-network methods | CNN, convolutional neural network | Multi-label |
| Primary goal | Diagnostic classification | Classification/differential diagnosis of dementia states | diagnosis, classification, discrimination, differential diagnosis | Single primary goal |
| Primary goal | Disease subtyping | Identification of disease heterogeneity or phenotypes | subtype, heterogeneity, phenotype, patient stratification, unsupervised learning | Single primary goal |

Abbreviations: ML, machine learning; APOE, apolipoprotein E; MRI, magnetic resonance imaging; CNN, convolutional neural network; Aβ, amyloid beta; p-tau, phosphorylated tau; DTI, diffusion tensor imaging; VBM, voxel-based morphometry

**Table S2.** Top 10 source journals for original research on machine learning in dementia.

| **Rank** | **Journal** | **Publications, N** | **% of original research*** |
| --- | --- | --- | --- |
| 1 | Scientific Reports | 111 | 3.44 |
| 2 | Journal of Alzheimer’s Disease | 104 | 3.23 |
| 3 | Frontiers in Aging Neuroscience | 88 | 2.73 |
| 4 | Biomedical Signal Processing and Control | 57 | 1.77 |
| 5 | PLOS ONE | 57 | 1.77 |
| 6 | IEEE Access | 56 | 1.74 |
| 7 | Diagnostics | 43 | 1.33 |
| 8 | Alzheimer’s Research & Therapy | 36 | 1.12 |
| 9 | Alzheimer’s & Dementia | 34 | 1.05 |
| 10 | Frontiers in Neuroscience | 32 | 0.99 |

Abbreviations: N, number; IEEE, Institute of Electrical and Electronics Engineers

**Note:** *Percentages are based on the 3,223 original research publications included in the thematic analysis. Journal names were derived from the Web of Science source-title field. Publications from 2026 were included through September 1, 2026.

**Table S3.** Top 10 contributing countries in original research on machine learning in dementia.

| **Rank** | **Country/region** | **Publications, N** | **% of original research*** |
| --- | --- | --- | --- |
| 1 | China | 776 | 24.1 |
| 2 | United States | 772 | 24.0 |
| 3 | India | 449 | 13.9 |
| 4 | England | 246 | 7.6 |
| 5 | South Korea | 209 | 6.5 |
| 6 | Saudi Arabia | 162 | 5.0 |
| 7 | Italy | 151 | 4.7 |
| 8 | Spain | 124 | 3.8 |
| 9 | Australia | 110 | 3.4 |
| 10 | Canada | 110 | 3.4 |

Abbreviations: N, number

**Note:** *Percentages are based on the 3,223 original research publications included in the thematic analysis. Countries were identified from author affiliation information. A publication involving authors from more than one country was attributed to each represented country; therefore, percentages are not mutually exclusive and do not sum to 100%. Publications from 2026 were included through September 1, 2026.

**Table S4.** Top contributing authors and institutions in original research on machine learning in dementia.

**Panel A.** Top 10 contributing authors.

| **Rank** | **Author** | **Publications, N** | **% of original research*** |
| --- | --- | --- | --- |
| 1 | Liu Y. | 27 | 0.84 |
| 2 | Zhang Y. | 26 | 0.81 |
| 3 | Wang L. | 24 | 0.74 |
| 4 | Wang Y. | 20 | 0.62 |
| 5 | Ramírez J. | 20 | 0.62 |
| 6 | Górriz J.M. | 19 | 0.59 |
| 7 | Kim S. | 18 | 0.56 |
| 8 | Li J. | 18 | 0.56 |
| 9 | Kim J. | 18 | 0.56 |
| 10 | Wang J. | 18 | 0.56 |

Abbreviations: N, number

**Panel B.** Top 10 contributing institutions.

| **Rank** | **Institution** | **Publications, N** | **% of original research*** |
| --- | --- | --- | --- |
| 1 | University of London | 73 | 2.26 |
| 2 | University of California System | 69 | 2.14 |
| 3 | Chinese Academy of Sciences | 57 | 1.77 |
| 4 | Egyptian Knowledge Bank | 57 | 1.77 |
| 5 | Harvard University | 55 | 1.71 |
| 6 | Harvard University Medical Affiliates | 47 | 1.46 |
| 7 | State University System of Florida | 46 | 1.43 |
| 8 | University of Pennsylvania | 46 | 1.43 |
| 9 | Capital Medical University | 41 | 1.27 |
| 10 | University of Texas System | 38 | 1.18 |

Abbreviations: N, number

**Table S5.** Top 10 highly cited original research publications on machine learning in dementia.

| **Rank** | **First author** | **Article title** | **Journal/Source** | **Year** | **WoS citations*** |
| --- | --- | --- | --- | --- | --- |
| 1 | Moradi et al. | Machine learning framework for early MRI-based Alzheimer’s conversion prediction in MCI subjects | *NeuroImage* | 2015 | 544 |
| 2 | Wen et al. | Convolutional neural networks for classification of Alzheimer’s disease: Overview and reproducible evaluation | *Medical Image Analysis* | 2020 | 534 |
| 3 | Basaia et al. | Automated classification of Alzheimer’s disease and mild cognitive impairment using a single MRI and deep neural networks | *NeuroImage: Clinical* | 2019 | 504 |
| 4 | Venugopalan et al. | Multimodal deep learning models for early detection of Alzheimer’s disease stage | *Scientific Reports* | 2021 | 463 |
| 5 | Ding et al. | A deep learning model to predict a diagnosis of Alzheimer disease by using 18F-FDG PET of the brain | *Radiology* | 2019 | 413 |
| 6 | Liu et al. | A multi-model deep convolutional neural network for automatic hippocampus segmentation and classification in Alzheimer’s disease | *NeuroImage* | 2020 | 387 |
| 7 | Gray et al. | Random forest-based similarity measures for multi-modal classification of Alzheimer’s disease | *NeuroImage* | 2013 | 359 |
| 8 | Magnin et al. | Support vector machine-based classification of Alzheimer’s disease from whole-brain anatomical MRI | *Neuroradiology* | 2009 | 346 |
| 9 | Liu et al. | Early diagnosis of Alzheimer’s disease with deep learning | *2014 IEEE 11th International Symposium on Biomedical Imaging* | 2014 | 332 |
| 10 | de Haan et al. | Functional neural network analysis in frontotemporal dementia and Alzheimer’s disease using EEG and graph theory | *BMC Neuroscience* | 2009 | 329 |

Abbreviations: WoS, Web of Science; MRI, magnetic resonance imaging; MCI, mild cognitive impairment; FDG, fluorodeoxyglucose; PET, positron emission tomography; EEG, electroencephalography; IEEE, Institute of Electrical and Electronics Engineers; BMC, BioMed Central

**Table S6.** Thematic interpretation of keyword co-occurrence clusters in machine learning-based dementia research.

| **Cluster** | **Keywords, N** | **Thematic interpretation** | **Most frequent keywords (occurrences)** | **Biomarker/biological terms represented** |
| --- | --- | --- | --- | --- |
| 1 | 102 | Clinical risk, biomarkers, and broader AI applications | Dementia (955); machine learning (954); artificial intelligence (257); disease (223); biomarkers (210); risk (155); brain (130); cognitive impairment (116); association (94); decline (72); prevalence (64); memory (64); older adults (63) | Biomarkers (210); biomarker (58); tau (57); amyloid beta (50); genetics (21); blood (18); CSF biomarkers (18); neuroinflammation (17) |
| 2 | 80 | Deep learning, neuroimaging, and diagnostic classification | Alzheimer’s disease (2,101); deep learning (754); classification (677); diagnosis (541); MRI (420); convolutional neural network (342); magnetic resonance imaging (283); transfer learning (167); neuroimaging (115); early diagnosis (117); feature extraction (100); PET (92); EEG (88) | PET (92); EEG (88); genetic algorithm (15) |
| 3 | 36 | MCI conversion, progression, and feature-based prediction | Mild cognitive impairment (851); prediction (275); atrophy (135); feature selection (123); progression (122); MCI (113); support vector machine (110); conversion (109); random forest (84); functional connectivity (69); structural MRI (69); hippocampus (63) | Atrophy (135); hippocampus (63); FDG-PET (60); brain atrophy (33); hippocampal (24); amyloid PET (15) |
| 4 | 10 | Diagnostic criteria, guidelines, and staging concepts | National Institute (151); recommendations (111); association workgroups (93); diagnostic guidelines (89); models (51); guidelines (41); Mini-Mental State (39); Alzheimer’s Association workgroups (30) | Hippocampal atrophy (19) |
| 5 | 4 | Frontotemporal dementia and differential diagnosis | Frontotemporal dementia (58); neurodegenerative diseases (30); behavioral variant (20); differential diagnosis (18) | No prominent biomarker-specific terms detected |

Abbreviations: N, number; AI, artificial intelligence; MCI, mild cognitive impairment; PET, positron emission tomography; EEG, electroencephalography; MCI, mild cognitive impairment; MRI, magnetic resonance imaging; CSF, cerebrospinal fluid; FDG, fluorodeoxyglucose

**Supplementary Figures**

**Figure S1.** Biomarker temporal trends.

**Figure S2.** Machine learning method × prediction-goal heatmap.

**Figure S3.** Machine learning method × data-modality heatmap.

**Figure S4.** Shared datasets/consortia.

**Figure S5.** Large language model/transformer temporal trends.
